# Preventing mental illness in children that experienced maltreatment the efficacy of REThink online therapeutic game

**DOI:** 10.1038/s41746-023-00849-0

**Published:** 2023-06-05

**Authors:** Oana A. David, Liviu A. Fodor

**Affiliations:** 1 DATA Lab, International Institute for the Advanced Studies of Psychotherapy and Applied Mental Health, Babeș-Bolyai University, Cluj-Napoca, Romania; 2 Department of Clinical Psychology and Psychotherapy, Babeș-Bolyai University, Cluj-Napoca, Romania

**Keywords:** Social sciences, Psychology

## Abstract

Exposure to child maltreatment (CM) is considered to predispose children to devastating consequences in terms of mental health. Thus, it is a public health priority to provide these children with early preventive interventions that are accessible on a large scale, adapted to their needs, and effective in supporting their mental health. Here we report a randomized control trial to test the efficacy of the REThink online therapeutic game, as compared with a Care as Usual (CAU) control group in the prevention of mental illness in maltreated children. Out of 439 children aged 8–12 that were recruited, 294 children with self-reported maltreatment histories were included in the current study, and were allocated, 146 participants in the REThink group and 148 participants in the CAU group. All children completed pre- and post-intervention assessments measuring mental health, emotion regulation, and irrational cognitions. We also tested potential moderators for these effects, such as the severity of CM and the security of parent attachment. Our results show that children receiving the REThink game intervention outperform the CAU group at post-test, showing a significantly lower level of emotional problems, mental health difficulties, use of maladaptive emotion-regulation strategies such as catastrophizing, rumination, and self-blame, and irrational cognitions. Moreover, children with higher CM severity benefit the most from the REThink game, while children with lower parent attachment security benefit the least. Future research is needed, to investigate the long-term efficacy of the REThink game in promoting the mental health of children exposed to CM.

## Introduction

Childhood maltreatment (CM), often referred to as childhood abuse and neglect (CAN), includes deprivation experiences, such as physical neglect, emotional neglect, or threatening experiences that occur during childhood, such as physical abuse, emotional abuse, or sexual abuse^1^. In terms of prevalence rates, it was estimated that almost a quarter (23%) of adults report having experienced physical abuse as children, and over one third (37%) report having been emotionally abused^2^. A large body of research has identified CM appearing at any stage of development to predispose children to devastating consequences on physical and mental health, and to adult psychopathology in the long run, regardless of culture or geographical variations. Multiple longitudinal studies^3^ have demonstrated that CM is associated with higher rates of mood disorders (i.e., 46% of individuals with depression report CM), earlier onset, persistence and increases in emotional disorders severity. Indeed, a recent review^4^ suggests that CM produces persistent alterations in the stress response systems and predispose children to enhanced threat detection, heightened emotionality and poor emotion regulation, which in turn are linking childhood adversity with psychopathology. Thus, early and accessible interventions are needed that can improve the mental health outcomes of the youths with a history of CM, by targeting these putative mechanisms.

Serious or therapeutic video games have been developed more recently and proved effective for approaching mental health concerns in children and adolescents^5^. REThink is such an online therapeutic game with a transdiagnostic approach to emotional disorders in youths, which was developed based on a tested cognitive-behavioral therapy (CBT) prevention protocol (Rational Emotive Behavior Education – REBE)^6^. The therapeutic video game has an action and adventure approach on the therapeutic tasks offered, with seven levels focused each on practicing a specific component of the therapeutic protocol in simulated situations. The main focus of the game is to provide an accessible and attractive instrument that can train emotion recognition abilities, relaxation and mindfulness skills, cognitive change, and to cultivate positive emotions and self-compassion in youths.

The game proved its efficacy in improving mental health of children and adolescents aged between 10–16 years, compared to a standard face to face cognitive-behavioral program and a waitlist^7,8^. Both short-term and 6-month follow-up results showed that children in the group reported medium size significant reduction in emotional symptoms, conduct problems and improvements in pro-social behavior. More specifically, youths that participated in the REThink game intervention reported significant improvements in their depressive mood, fear, attention, inhibitory control and emotion-regulation competencies (emotional self-awareness and emotional control), as compared to the wait-list group. The mechanisms of change analyses^9^ showed that cognitive changes (i.e., irrational cognitions) mediated changes in the REThink intervention, in accordance with the cognitive-behavioral theory on which the game is based. Furthermore, it was found^10^ that the REThink game can improve biological reactivity to stress of the children with changes that are maintained at 6-month follow-up.

The present research therefore aimed to test if the REThink therapeutic game is effective as a standalone intervention for improving the mental health and emotion-regulation abilities for children and adolescents that experienced CM. We hypothesized that the participants in the REThink therapeutic game will report similar robust improvements in these outcomes to those documented in the previous trials^7–12^, relative to the control condition after the intervention. Based on previous findings on moderators for the treatment efficacy showing that CM is associated with poor treatment outcomes^13^, we expected that improvements in the outcomes for the REThink intervention will be lower for the youths presenting with higher severity of CM, or with additional risk factors such as insecure parent attachment.

## Results

### Sample characteristics

There were no statistically significant differences between the participants in the REThink group and the participants in the CAU group in terms of gender, *χ*^2^(1, *N* = 294) = 0.01, *p* = 0.903 or age, *t*(292) = 542, *p* = 0.588.

### Multivariate and mixed model analyses

With regard to the multivariate ANOVA main analysis, the within-subjects time effect (pre- vs. post-intervention) was statistically non-significant, Pillai’s Trace = 0.10, *F*(20, 216) = 1.28, *p* = 0.193. However, we identified a statistically significant between-subjects group effect (REThink vs. CAU), Pillai’s Trace = 0.13, *F*(20, 216) = 1.64, *p* = 0.045, *η*^*2*^*p* = 0.13. Moreover, we also identified a statistically significant interaction effect (time × group effect), Pillai’s Trace = 0.13, *F*(20, 216) = 1.72, *p* = 0.031, *η*^*2*^*p* = 0.13.

Following the statistically significant multivariate effects with separate linear mixed models analyses, statistically significant univariate group effects were identified for conduct problems - *F*(1, 212.56) = 4.31, *p* = 0.039, emotional self-awareness - *F*(1, 231.77) = 5.19, *p* = 0.024, intolerance to frustrating rules - *F*(1, 223.74) = 6.72, *p* = 0.010 and intolerance to work frustration - *F*(1, 244.42) = 4.12, *p* = 0.043. Post-hoc Bonferroni-adjusted pairwise comparisons revealed that, averaged over time, participants in the REThink group had lower scores than the participants in the CAU group for conduct problems, intolerance to frustrating rules and intolerance to work frustration, while participants in the REThink group had higher scores than the participants in the CAU group for emotional self-awareness.

Furthermore, linear mixed models analyses revealed statistically significant univariate time x group interaction effects for eight dependent variables, namely for emotional problems - *F*(1, 139.25) = 12.78, *p* < 0.001, total difficulties - *F*(1, 135.74) = 7.14, *p* = 0.008, emotional control - *F*(1, 150.21) = 4.28, *p* = 0.040, self-downing - *F*(1, 148.82) = 15.64, *p* < 0.001, total irrationality - *F*(1, 137.64) = 12.69, *p* = 0.001, rumination - *F*(1, 158.93) = 6.21, *p* = 0.014, catastrophizing - *F*(1, 150.06) = 17.28, *p* < 0.001 and self-blame - *F*(1, 142.78) = 5.83, *p* = 0.017.

For emotional problems and total difficulties, post-hoc Bonferroni-adjusted pairwise comparisons revealed that the interaction effect was explained both by a decrease in scores in the REThink group from pre- to post-intervention (*p* < 0.05 for both outcomes, *d* = 0.35 for emotional problems and *d* = 0.22 for total difficulties) and by a statistically significant difference between the REThink and CAU groups at post-intervention, participants in the REThink group registering lower scores compared to participants in the CAU group for both emotional problems (*p* = 0.001, *d* = 0.42) and total difficulties (*p* = 0.007, *d* = 0.35).

In the case of emotional control, post-hoc Bonferroni-adjusted pairwise comparisons revealed that the interaction effect was due to a statistically significant increase in scores from pre- to post-intervention in the CAU group (*p* = 0.025) and by a preexisting difference between groups at pre-intervention (*p* = 0.005). As a difference between groups was identified at pre-intervention, we employed a univariate ANCOVA analysis comparing post-intervention scores between groups, with the pre-test scores as covariates. The ANCOVA analysis revealed no statistically significant differences between groups, *F*(1, 122) = 1.30, *p* = 0.256.

With regard to rumination, post-hoc Bonferroni-adjusted pairwise comparisons revealed that the interaction effect was explained both by an increase in scores from pre- to post-intervention in the CAU group (*p* = 0.017, *d* = 0.30) and a statistically significant difference between groups at post-intervention, the REThink group registering lower scores compared to the CAU group (*p* = 0.019, *d* = 0.31). Similarly, in the case of catastrophizing, post-hoc Bonferroni-adjusted pairwise comparisons revealed that the interaction effect was due to modifications from pre- to post-intervention, albeit in both groups, with scores decreasing in the REThink group (*p* = 0.001, *d* = 0.34) and increasing in the CAU group (*p* = 0.009, *d* = 0.29). Moreover, there was a statistically significant difference between groups at post-intervention, the REThink group participants reporting lower scores than the CAU group (*p* = 0.002, *d* = 0.41). Finally, in the case of self-blame, post-hoc Bonferroni-adjusted pairwise comparisons revealed that the interaction effect was due to a statistically significant difference between groups at post-intervention, the REThink group registering lower scores compared to the CAU group (*p* = 0.017, *d* = 0.26).

For self-downing, post-hoc Bonferroni-adjusted pairwise comparisons revealed that the interaction effect was explained by statistically significant modifications in scores from pre- to post-intervention, with scores decreasing in the REThink group (*p* = 0.004, *d* = 0.25) and increasing in the CAU group (*p* = 0.008, *d* = 0.26). Moreover, there was a statistically significant difference between the REThink and CAU groups at post-intervention, participants in the REThink group registering lower scores than participants in the CAU group (*p* < 0.001, *d* = 0.51). In the case of total irrationality, post-hoc Bonferroni-adjusted pairwise comparisons revealed that the interaction effect was explained both by a decrease in scores in the REThink group from pre- to post-intervention (*p* < 0.001, *d* = 0.34) and by a statistically significant difference between the REThink and CAU groups at post-intervention, participants in the REThink group showing lower scores than participants in the CAU group (*p* < 0.001, *d* = 0.54).

All estimated marginal means, together with their standard errors, for all outcomes are detailed in Table 1.

**Table 1:** Descriptive statistics: Estimated marginal means (M) and standard errors (SE) for each group, at each time point, for all dependent variables.

	CAU	REThink				
*N*	148	146				
Age	10.07 (0.07)	9.99 (0.07)	*t* (292) = 542, *p* = 0.588			
Gender (% females)	52 %	52.7 %	χ^2^(1, *N* = 294) = 0.01, *p* = 0.903			
CTQ-SF						
Emotional abuse	7.63 (0.31)	6.52 (0.20)				
Physical abuse	6.26 (0.19)	5.84 (0.12)				
Emotional neglect	7.63 (0.33)	6.94 (0.23)				
Physical neglect	6.49 (0.19)	6.20 (0.13)				
Total trauma	39.74 (0.46)	37.70 (0.27)				
Multivariate ANOVA test for between-group difference in trauma scores	Pillai’s trace = 0.03, *F* (5, 266) = 1.93, *p* = 0.088					

	Pre-intervention (M, SE)			Post-intervention (M, SE)		
	CAU (*N* = 148)	REThink (*N* = 146)	MD (95% CI)	CAU (*N* = 117)	REThink (*N* = 131)	MD (95% CI)
SDQ						
Emotional problems	3.08 (0.19)	3.09 (0.19)	−0.12 (−0.55 to 0.52)	3.42 (0.27)	2.22 (0.24)	1.20 (0.47 to 1.92)
Conduct problems	2.74 (0.11)	2.58 (0.11)	0.16 (−0.16 to 0.48)	3.09 (0.18)	2.52 (0.16)	0.57 (0.08 to 1.06)
Hyperactivity	5.41 (0.14)	5.33 (0.14)	0.07 (−0.32 to 0.46)	5.19 (0.21)	5.15 (0.18)	0.03 (−0.53 to 0.60)
Peer problems	4.73 (0.10)	4.68 (0.10)	0.05 (−0.25 to 0.35)	4.84 (0.15)	4.54 (0.13)	0.29 (−0.11 to 0.69)
Prosocial behavior	8.31 (0.14)	8.57 (0.14)	−0.25 (−0.65 to 0.14)	8.14 (0.22)	8.42 (0.19)	−0.28 (−0.87 to 0.29)
Total difficulties	15.98 (0.40)	15.68 (0.41)	0.30 (−0.84 to 1.44)	16.60 (0.57)	14.46 (0.52)	2.13 (0.60 to 3.67)
ERICA						
Emotional self-awareness	18.77 (0.39)	20.17 (0.35)	−1.40 (−2.45 to −0.35)	19.13 (0.32)	19.65 (0.29)	−0.51 (−1.38 to 0.34)
Emotional control	25.42 (0.75)	28.30 (0.66)	−2.87 (−4.86 to −0.89)	27.09 (0.63)	27.95 (0.58)	−0.86 (−2.56 to 0.83)
CASI						
Self-downing	16.99 (0.56)	16.82 (0.54)	0.17 (−1.38 to 1.72)	18.73 (0.67)	15.15 (0.59)	3.58 (1.81 to 5.35)
Intolerance of frustrating rules	12.87 (0.44)	11.84 (0.42)	1.02 (−0.18 to 2.23)	13.38 (0.58)	11.25 (0.52)	2.12 (0.57 to 3.67)
Intolerance of work frustration	23.50 (0.49)	22.78 (0.47)	0.71 (−0.63 to 2.06)	22.83 (0.60)	20.90 (0.54)	1.93 (0.32 to 3.53)
Demands for fairness	18.77 (0.32)	18.65 (0.31)	0.12 (−0.77 to 1.01)	18.39 (0.45)	17.23 (0.39)	1.15 (−0.32 to 2.35)
Total irrationality	72.40 (1.37)	69.99 (1.32)	2.41 (−1.34 to 6.16)	73.67 (1.60)	64.60 (1.43)	9.06 (4.82 to 13.31)
CERQ-k						
Acceptance	6.19 (0.17)	6.34 (0.16)	−0.15 (−0.62 to 0.32)	6.62 (0.25)	6.70 (0.21)	−0.08 (−0.74 to 0.57)
Positive reappraisal	6.59 (0.18)	6.72 (0.17)	−0.13 (−0.63 to 0.36)	6.68 (0.27)	6.36 (0.24)	0.32 (−0.39 to 1.05)
Rumination	5.64 (0.19)	5.83 (0.17)	−0.18 (−.69 to 0.32)	6.40 (0.27)	5.54 (0.23)	0.86 (0.14 to 1.58)
Positive refocusing	5.82 (0.22)	6.13 (0.21)	−30 (−0.92 to 0.30)	5.64 (0.32)	6.27 (0.28)	−0.62 (−1.47 to 0.22)
Catastrophizing	4.06 (0.18)	4.46 (0.17)	−0.40 (−0.89 to 0.09)	4.74 (0.24)	3.70 (0.21)	1.03 (0.38 to 1.68)
Refocus on planning	7.20 (0.19)	7.40 (0.18)	−0.19 (−0.72 to 0.32)	7.17 (0.28)	7.16 (0.24)	0.01 (−0.72 to 0.75)
Putting into perspective	6.06 (0.21)	6.25 (0.20)	−0.18 (−0.77 to 0.40)	6.46 (0.30)	6.64 (0.26)	−0.17 (−0.97 to 0.61)
Self-blame	3.76 (0.15)	3.91 (0.14)	−0.15 (−0.58 to 0.27)	4.14 (0.23)	3.51 (0.20)	0.62 (0.01 to 1.24)
Other-blame	3.30 (0.15)	3.53 (0.14)	−0.22 (−0.63 to 0.18)	3.30 (0.20)	3.18 (0.17)	0.12 (−0.40 to 0.65)

REThink = experimental intervention group; MD = Bonferroni-adjusted post-hoc between-group mean difference; 95% CI = 95% confidence interval for the Bonferroni-adjusted post-hoc between-group mean difference.

*CTQ-SF* Childhood Trauma Questionnaire - Short Form, *SDQ* Strengths and Difficulties Questionnaire, *ERICA* Emotion-Regulation Index for Children and Adolescents, *CASI* Child and Adolescent Scale of Irrationality, *CERQ-k* Cognitive Emotion Regulation Questionnaire, *CAU* care-as-usual control group.

### Moderation analyses

Regarding potential moderation effects, we identified several group x moderator interactions, in case of emotional problems, total difficulties, and self-blame outcomes. Firstly, we identified a statistically significant interaction between the intervention group and total trauma scores, *F*(1, 255.07) = 5.01, *p* = 0.026, on the emotional problems outcome. Post-hoc Bonferroni-adjusted pairwise comparisons indicated that, averaged over time, an increase in total trauma scores corresponded to an increase of the difference between the REThink and CAU groups in terms of emotional problem scores, with the REThink group obtaining lower scores at post-intervention than the CAU group. Secondly, we identified a statistically significant interaction between the intervention group and total trauma scores, *F*(1, 255.76) = 5.89, *p* = 0.016, on the total difficulties outcome. Post-hoc Bonferroni-adjusted pairwise comparisons indicated that, averaged over time, an increase in total trauma scores corresponded to an increase of the difference between the REThink and CAU groups in terms of total difficulties scores, with the REThink group having lower scores at post-intervention than the CAU group. Thirdly, we have also identified a statistically significant interaction between the intervention group and security of parental attachment scores, *F*(1, 240.90) = 4.42, *p* = 0.037, on the self-blame outcome. Post-hoc Bonferroni-adjusted pairwise comparisons indicated that, averaged over time, an increase in security of parental attachment scores corresponded to an increase of the difference between the REThink and CAU Groups in terms of self-blame scores, with the REThink group having lower scores at post-intervention than the CAU group.

## Discussion

The aim of this study was to test the efficacy of the online REThink therapeutic game in improving the mental health and emotional-regulation abilities in children with CM.

In terms of CM, we observed a high prevalence of self-reported maltreatment (294 out of 439 screened participants). This could be explained by the fact that there is a high variability on reported maltreatment across cultures, with low-income countries reporting higher rates especially when it comes to physical abuse and neglect, including Romania (see^14^; in the Romanian population the incidence is 71.9% for physical and emotional forms of maltreatment). Moreover, data shows that during the COVID-19 pandemic the rates of child maltreatment have risen^15^ and this could also explain the high rates that were obtained in our sample.

As hypothesized, the results obtained show that the REThink intervention had brought statistically significant improvements compared to the CAU group at post-intervention in terms of emotional problems and mental health difficulties. Participants in the REThink intervention showed significantly better abilities for emotional control, compared to the CAU group which deteriorated in this matter. The participants in the REThink intervention outperformed the CAU group at post-test with regard to a significantly lower level of maladaptive emotion-regulation strategies employed, such as catastrophizing, rumination and self-blame. The magnitude of the changes is low to medium level for the strategies that improved significantly in the REThink intervention. This is a result that is extremely relevant for the CM youths, since it is documented that maladaptive emotion-regulation strategies are involved in the CM psychopathology link^4^.

The REThink intervention also had a positive impact with regard to cognitive changes. The children participating in the REThink intervention outperformed the CAU group with regard to irrational cognitions. The irrational cognitions scores of the children in the REThink intervention group significantly decreased to post-intervention with a medium size of improvements. This result gives support to the previously documented cognitive mechanism of change documented for the REThink intervention in our primary trial^8^.

Regarding potential moderators for differences between groups at post-test, we found significant group x moderator interactions for the severity of CM and the security of attachment to parents. First, CM severity was a significant moderator for between-group differences at post-test, with regard to both emotional problems and mental difficulties, but not in the expected direction based on the literature. Participants in the REThink group with higher CM severity registered significantly higher improvements in terms of emotional problems and mental health difficulties compared with the CAU group. This finding is however in line with our previous findings^8^ when the REThink intervention impact in reducing conduct problems in the long-term in general population of children and adolescents was higher for those with CM history. This finding shows great promises, given that CM has been consistently shown to be associated with poor treatment outcomes in youth depression, as assessed by lack of /response or longer time for remission^13^. Given the associations that were found^16^ for CM with alterations in reward-based systems, such as reinforcement and incentive-based learning (i.e., reward anticipation, and reward responsiveness), it might be that a game-based intervention, using closed incentivized based learning of emotional skills, supports also the regulation of reward-based systems during stress inducing context in the level 7 of the game. Future studies should investigate also reward-based mechanisms for the changes in children with CM.

Secondly, security of attachment to parents was a significant moderator for the differences in the usage of the self-blame emotion-regulation strategy between the groups. More specifically, children participating in the REThink Intervention with a higher level of security of parent attachment were able to significantly reduce the use of self-blame for regulating their emotions, compared to those with a lower level of security of parent attachment. This result is in line with previous findings^17^ suggesting that attachment security is a key factor in the relation between CM and the development of mood disorders, and family interventions are suitable for addressing this risk factor.

One limitation of the current study is that we used standard assessment points and we were not able to detect the dynamics of the changes in the variables during the trial and test potential mechanisms of change. Second, in this trial, we measured only the short-term efficacy of the REThink game for children with self-reported CM history and future studies need to document its long-term effects. Another limitation is the use of CTQ-SF in children, given that the measure was previously used with adolescents. This is why we considered only the total severity score and were not able to perform moderation per types of CAN and thus our findings pertaining to the moderation for the severity of the CM need to be viewed cautiously and future studies need to rely on other informants in this age range.

In terms of future research directions, the REThink game could be tested in a format which incorporates additional therapeutic personalized support. The game currently allows for features such as including personalized challenges set for the users, after each level, regarding the implementation of the skills that were learned during the game in real life. Also, we have studies in preparation that include testing the game in an adaptive design, using additional psychotherapeutic interventions (e.g., parent support) depending on the gains that are recorded.

## Methods

### Participants

**Fig. 1:**
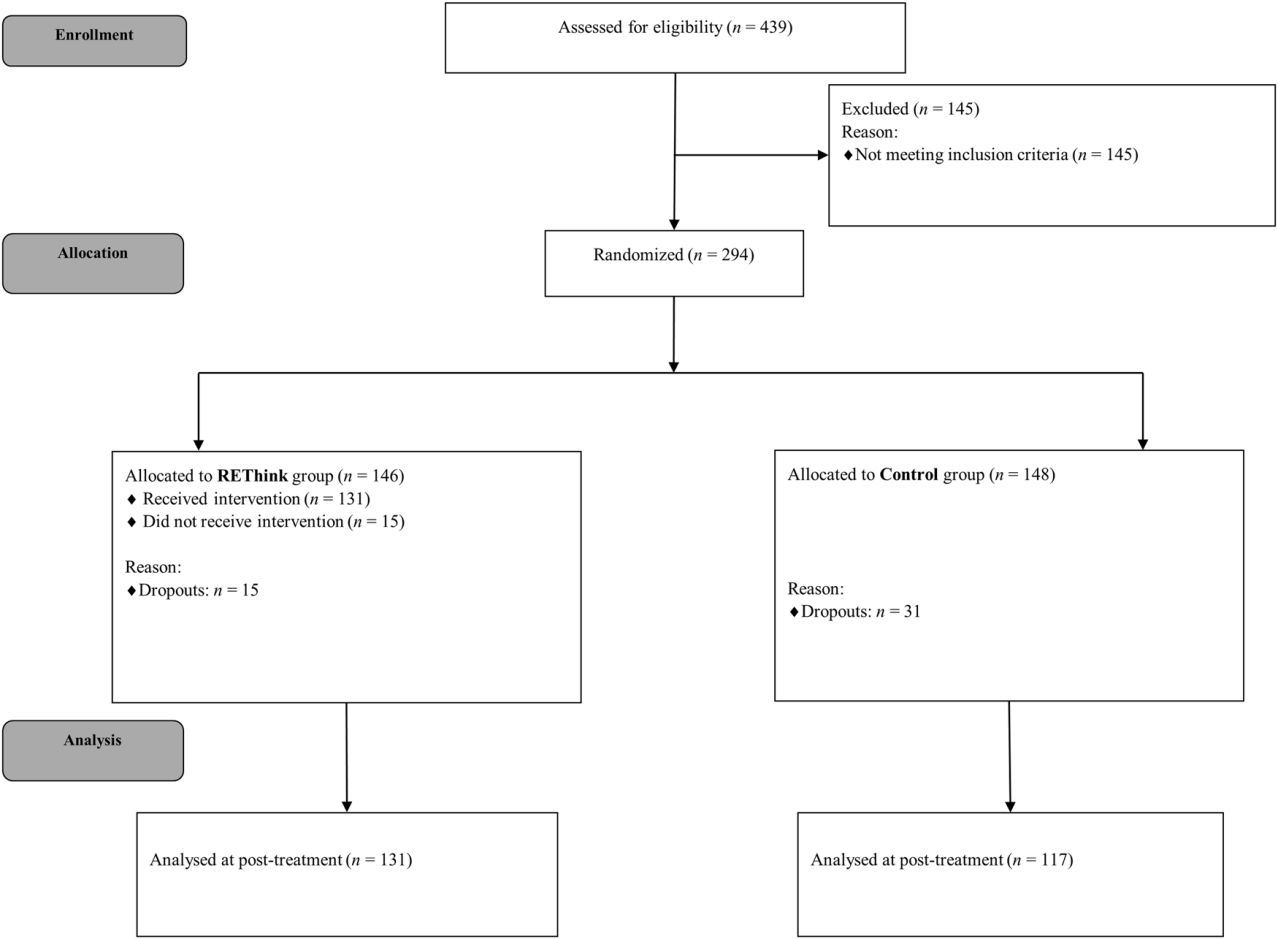
The CONSORT flow diagram. Participants’ progress throughout the phases of the trial, from enrollment to post-treatment.

439 students aged 8–12 years were recruited from 12 schools and completed the screening questionnaire, namely the four subscales of the Childhood Trauma Questionnaire - Short Form (CTQ-SF^18,19^), measuring dichotomically the presence of Emotional Neglect, Physical Neglect, Emotional Abuse, or Physical Abuse, by considering the items with responses different from ‘never true’. 294 children (M_age_ = 10.03‚ SD = 1.28) that presented with a self-reported maltreatment history were included in the current study, and were randomly allocated, 146 participants in the REThink group and 148 participants in the Care as Usual (CAU) control group. In the CAU group the percent of females was 52%, while in the REThink group the percent of females was 52.7% (Table 1). Figure 1 presents the flow of participants over the course of the study.

Baseline data were available for all 294 randomized. At post-test, data were available for 248 participants (84.35%), with 131/146 (89.72%) completing post-assessment in the REThink group, and 117/148 (79.05%) in the CAU group.

Of the children in the REThink game intervention group, 95 (65.07%) completed all 7 levels/sessions, 24 (16.44%) completed between 5 or 6 levels, and 27 (18.49%) completed fewer than 5 levels. Participants with fewer than 5 sessions are defined as dropouts from treatment.

There were no significant differences on demographic data or baseline measures between those remaining in treatment and those dropping out.

### Procedure

The study protocol was registered at ClinicalTrials.gov, with identifier NCT05424900 from May 3^rd^, 2021. Ethical approval for the study was obtained from the Ethics Committee of Babeș-Bolyai University, with registration number 2576, from March 18^th^, 2021. Informed consent (including processing of personal data according to EU regulations) to participate / to publish was obtained from the participants’ parents and from each school’s principal. The REThink game intervention was based on a protocol and its delivery was supervised by an experienced psychologist trained in CBT (first author). The contents of the intervention are briefly presented below, and additional details can be found in other published trials^7–12^.

### The REThink game

REThink is a therapeutic online game which was used in this study as a mobile application based on a transdiagnostic cognitive-behavioral program that reduces emotional symptoms and builds emotion-regulation competencies in children and adolescents. In the game, the user has the mission of helping Earth inhabitants to learn how to neutralize the irrational thinking influence (called the Bad mind) of the negative character, Irrationalizer, and instill the rational and compassionate thinking (called the Good mind), with the help of the rational character which guides the user, namely RETman.

**Fig. 2:**
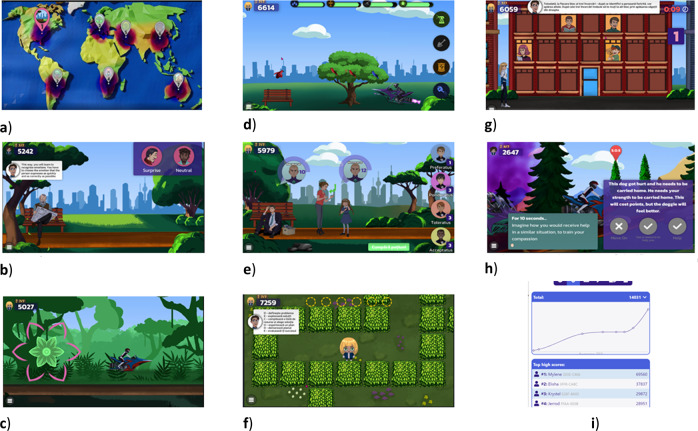
Illustration of the seven levels of the REThink game. **a** The mission, **b** emotion recognition, **c** relaxation and mindfulness, **d** effects of irrational cognitions, **e** neutralizing irrational cognitions, **f** problem-solving, **g** positive attention training, **h** practice during stress and (self) compassion, **i** platform with multiplayer.

By progressing throughout the seven levels of the game (see Fig. 2), the user learns and practices in simulated environments the following abilities: emotional recognition (Level 1), relaxation and mindfulness (Level 2), the relationship between beliefs and wellbeing (Level 3), neutralizing irrational beliefs with rational alternatives (Level 4), problem solving abilities (Level 5), positive attention retraining (Level 6) and practice of compassion, self-compassion, and practicing previously learned skills altogether during a stress inducing context (Level 7). In its current improved version, the game is offering self-compassion statements when the user fails and it offers the possibility to include behavioral challenges after each level regarding the application of the skills learned in real life, which was activated in the current study. Each level was played once by each participant, once per week, on a mobile device. At school, each participant registered an account in the game platform via email, and the game was played during one of the classes, under the supervision of a research assistant.

The game is promising, given the findings of the studies investigating its efficacy and effectiveness^7–12^ and it follows recommendations from current literature^20^. It is unique, given that there is a limited percentage of preventive games and that the game has a validated gamified assessment system^21^. Also, in line with the recommendations, the game was adapted over time to work on mobile devices and the graphics were improved. There is a clinician/researcher platform allowing the personalization of specific features, offering challenges for behavioral changes connected to the skills learned (as used in this trial) or sharing the scores with friends in a multi-player framework. Currently, the game is implemented both in a virtual reality and in a 3D format that will make it more attractive to adolescents and young adults.

### The care as usual control condition

The care as usual condition (CAU) participants took part only in the assessment phases during the trial.

### Measures

Participants completed assessment before the intervention (pre-test) and at the time of termination (post-test). The following primary outcomes were considered:

- Emotional symptoms, which were measured with the Strengths and Difficulties Questionnaire — child version (SDQ^22^). The SDQ was used to assess emotional symptoms as a primary outcome and, as secondary outcomes, the total level of psychological difficulties, conduct problems, hyperactivity, peer relationship problems, and prosocial behavior. SDQ is a 25-item questionnaire with responses on a three-point Likert scale, ranging from 0 “not true” to 2 “certainly true”. Lower scores lower scores in the problems subscales represent an improvement or lack of symptoms. The SDQ is documented to have adequate psychometric properties^23^, with a Cronbach’s alpha coefficient of 0.77 in our sample.
- Emotion regulation abilities were measured with the Emotion-Regulation Index for Children and Adolescents (ERICA^24^). We have assessed dimensions such as emotional control (i.e., socially adequate emotional expressions and responses with scores ranging from 5 to 25), and emotional self-awareness (i.e., emotional recognition and flexibility in regulating positive affect and negative affect, with scores ranging from 8 to 40). Higher scores reflect more adaptive or functional emotion-regulation abilities. ERICA has been shown to have good psychometric properties in previous studies^25^ and an alpha Cronback coefficient of 0.84 in our sample.
- Emotion regulation strategies were measured with the Cognitive Emotion Regulation Questionnaire (CERQ-k) short version^26^. The short form of the CERQ-k has 18 items, and each has answer categories ranging from 1 [(almost) never] to 5 [(almost) always]. CERQ-k was used it to measure nine emotion-regulation strategies employed by children during negative life events: self-blame, acceptance, rumination, positive refocusing, refocus on planning, positive reappraisal, putting into perspective, catastrophizing, and other-blame. CERQ-k has been shown to have good psychometric properties in previous studies^27^. Since CERQ-k short version does not provide a total score, we computed separate Cronbach’s alpha coefficients for each subscale: self-blame (0.41), acceptance (0.33), rumination (0.46), positive refocusing (0.78), refocus on planning (0.59), positive reappraisal (0.39), putting into perspective (0.67), catastrophizing (0.59) and other-blame (0.56).

With regard to secondary outcomes, the following were considered:

- Mental health symptoms (measured with SDQ and described above);
- The cognitive changes, expressed in irrational cognitions of children were measured using the Child and Adolescent Scale of Irrationality (CASI^28^). CASI is a 28- item scale that measures irrational cognitions defined as rigid beliefs that children and adolescents can have regarding relevant life domains. Items reflect irrational cognitions in several domains: demandingness for fairness (DEM-F), low frustration tolerance for work (LFT-W), low frustration tolerance of rules (LFT-R), and global evaluation (GE) of self and others. Children are asked in the scale to mark their agreement/disagreement with the 28 statements based on a 5-point Likert scale, from 1 (strong disagreement) to 5 (strong agreement). The CASI has adequate psychometric properties^29^ and has registered an alpha Cronbach coefficient of 0.88 in our sample.

With regard to potential moderating variables for changes in the outcomes, the following were considered:

- Childhood maltreatment was measured with the Childhood Trauma Questionnaire - Short Form (CTQ-SF^18,19^), making use of the following subscales: Emotional Neglect, Physical Neglect, Emotional Abuse, or Physical Abuse, consisting each of five items. The CTQ-SF is a self-reported screening instrument measure of childhood maltreatment in children and adolescents. The four abuse and neglect subscales used use sums of the scorings from ‘never true’ (score 1) to ‘very often true’ (score 5), with seven reversed items. We computed the Total Trauma score as a sum of the four used subscales. The measure has adequate psychometric properties (internal consistency of 0.89 for the total scale^19^). We obtained an alpha Cronbach of 0.84 for total severity score in our sample while, for the subscales that we used, Cronbach’s alpha coefficients were the following: 0.74 for emotional *abuse*, 0.60 for physical abuse, 0.82 for emotional neglect and 0.30 for physical neglect.
- The security of parental attachment was measured with the Security Scale (SS^30^). The SS is used to assess children’s perceptions of security in parent-child relationships in children aged 9–14. The scale has 15 items that measures the following dimensions: the degree to which children believe that the attachment figure is responsive and available (e.g., whether a child worries that a parent will not be there when needed); the child’s intention to rely on the attachment figure in times of stress (e.g., whether a child goes to a parent when upset); and child’s reported interest in disclosing with the attachment figure (e.g., whether a child likes to tell a parent what she or he is thinking and feeling). Each item is scored on a 4-point scale, with higher scores indicating a more secure attachment, and scores across items are averaged to extract a score on a continuous dimension of security. This version of the scale has demonstrated adequate internal consistency of 0.74 or higher (^31,32^), while in our sample, we obtained a Cronbach’s alpha coefficient of 0.86.

### Statistical analysis

Firstly, in order to reduce potential Type I error rates associated with multiple outcome testing, we initially employed a 2 (intervention group: REThink, CAU) × 2 (assessments: pre-intervention, post-intervention) multivariate analysis of variance (MANOVA) to estimate the main effects and potential interactions. Statistically significant overall multivariate main effects and interactions were pursued by employing separate univariate mixed models analyses (LMM) for each dependent variable, while allowing for random participant intercepts. We employed the LMM approach for two main reasons, namely because (1) the inclusion of random participant intercepts accounts better for the timepoint to timepoint dependency of the data that is characteristic for repeated measures and (2) allows for the inclusion of more datapoints by not performing listwise deletion in those cases when there are missing observations at one timepoint, thus maximizing statistical power and being better suited for modeling changes over time^33^. Finally, a diagonal repeated covariance matrix type was employed (i.e., the default covariance structure for repeated effects), and statistically significant main effects and interactions were followed by pairwise comparisons of estimated marginal means using the Bonferroni correction for multiple comparisons. With regard to indicators of effect size, for the multivariate main effects and interactions we computed *η*^*2*^*p*, while for statistically significant pairwise comparisons we computed Cohen’s *d*. For outcomes where pre-existing differences at pre-intervention between groups were observed, we employed a univariate analysis of covariance (ANCOVA), comparing post-intervention scores between groups, with the pre-test scores as covariates.

Secondly, in order to estimate potential moderator effects, we checked for statistically significant interactions between intervention group type and the specific moderators, as averaged over time (i.e., from pre-intervention to post-intervention). When specific cut-off scores were not provided, moderators were dichotomized according to the 25^th^, 50^th^ and 75^th^ percentiles. All analyses were performed using SPSS version 26^34^ and Jamovi version 2.3.18^35^ statistical software.

### Dropout

Of the total sample of 294 children included in this study (146 participants in the REThink group and 148 participants in the CAU group), 248 participants completed the post-test assessment (see Fig. 1).

## Data Availability

The data that support the findings of this study are available from the corresponding author, upon reasonable request.

## Code availability

No custom code was used in this study.
