# Author Correction: Preventing mental illness in children that experienced maltreatment the efficacy of REThink online therapeutic game

**DOI:** 10.1038/s41746-023-00984-8

**Published:** 2023-12-14

**Authors:** Oana A. David, Liviu A. Fodor

**Affiliations:** 1 DATA Lab, International Institute for the Advanced Studies of Psychotherapy and Applied Mental Health, Babeș-Bolyai University, Cluj-Napoca, Romania; 2 Department of Clinical Psychology and Psychotherapy, Babeș-Bolyai University, Cluj-Napoca, Romania

**Keywords:** Social sciences, Psychology

Correction to: *npj Digital Medicine* 10.1038/s41746-023-00849-0, published online 05 June 2023

In the Acknowledgements section, the funding source ‘This work was supported, in terms of funding, by two grants awarded to O.A.D. from the Romanian National Authority for Scientific Research, CNCS—UEFISCDI (grant numbers PN-II-PT-PCCA2013-4-1937 and PN-III-P2-2.1-PED-2019-3837)’. should have read ‘This work was supported by two grants awarded to the first author from the Romanian National Authority for Scientific Research, CNCS—UEFISCDI (Grant number PN-III-P2-2.1-PED-2019-3837 and PN-III-P4-ID-PCE-2020-2170).’ The original article has been corrected.

